# Association of Acute Interstitial Nephritis with Carnivora, a Venus Flytrap Extract, in a 30-Year-Old Man with Hodgkin's Lymphoma

**DOI:** 10.1155/2014/486173

**Published:** 2014-04-15

**Authors:** Susan Ziolkowski, Catherine Moore

**Affiliations:** University of Rochester Medical Center, 601 Elmwood Avenue, P.O. Box MED, Rochester, NY 14642, USA

## Abstract

Acute interstitial nephritis (AIN) is a common cause of acute kidney injury and has been associated with a variety of medications. This is the case of 30-year-old man with Hodgkin's lymphoma who on routine labs before chemotherapy was found to have acute nonoliguric renal failure. A kidney biopsy was performed and confirmed the diagnosis of acute interstitial nephritis. The patient had taken several medications including a higher dose of Carnivora, a Venus flytrap extract, composed of numerous amino acids. The medication was discontinued and kidney function improved towards the patient's baseline indicating that this may be the possible cause of his AIN. Proximal tubular cell uptake of amino acids increasing transcription of nuclear factor-kappaB is a proposed mechanism of AIN from this compound.

## 1. Introduction

Acute interstitial nephritis (AIN) is a common cause of acute kidney injury (AKI) causing immune-mediated tubulointerstitial injury with an overall incidence of 15–27% of all renal biopsies performed for AKI [1]. Medications, most notably NSAIDs and antibiotics, are the most common cause of AIN representing >75% of cases. Systemic diseases such as sarcoidosis and systemic lupus erythematosus are other causes, while about 5–10% of cases are idiopathic [1]. Cases of AIN commonly present with nonspecific signs such as nausea, vomiting, and malaise. Other findings such as fever, rash, eosinophilia, oliguria, and arthralgia present at varying frequencies [2]. Renal manifestations of AIN generally occur within three weeks of the inciting drug with an average delay of ten days [3].

Patients who discontinue the inciting medication within two weeks of the onset of AIN are more likely to recover renal function than those who continue the medication for a longer period of time [4]. Therefore, new medications that are thought to cause AIN should be reported and promptly discontinued by a clinician to improve a patient's chance of recovery.

Carnivora is a supplement that is extracted from* Dionaea muscipula*, a species of the Venus flytrap plant. The product is manufactured in Germany, sold via the internet, and marketed as an “immune modulator” and antioxidant that selectively kills “primitive cells” that intrude the human body without harming the body's own cells. The physiologic effect is much like the Venus flytrap where foreign animal and vegetable products are digested without harming the plant's own cells. Numerous compounds including droserone, hydroplumbagin, quercetin, formic acid, myricetin, gallic acid, and several amino acids are naturally present in this compound. There have been no reported cases of AIN associated with Carnivora, exposure to Venus flytraps, or any of the above compounds [5]. The patient presented had other types of drug exposure such as metoclopramide, promethazine, and baclofen; however, a likely association with Carnivora is discussed below.

## 2. A Case Report

**Figure 1:**
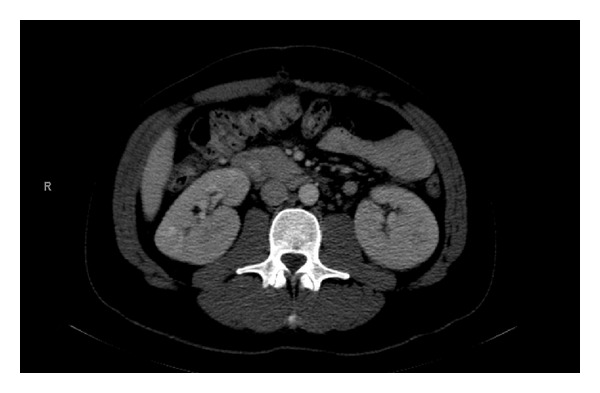
CT scan with oral contrast showing enlarged 15 cm right kidney and 14.5 cm left kidney with no hydronephrosis.

The patient is a 30-year-old man with recurrent Hodgkin's lymphoma originally diagnosed in January 2010 who underwent an autologous stem cell transplant at that time. Routine restaging scans revealed a recurrence in abdominal lymph nodes and the patient underwent an exploratory laparotomy and mesenteric lymph node biopsy, which confirmed a recurrence of Hodgkin lymphoma. During this hospitalization, he had two doses of ampicillin-sulbactam and limited doses of promethazine, metoclopramide, and baclofen. He admitted to taking a supplement, Carnivora, at home for about one year, which he discontinued during his hospital stay. He was discharged from the hospital with a creatinine of 0.94 mg/dL. After leaving the hospital, he resumed Carnivora at a higher dose (4 capsules, three times a day). Four weeks later, the creatinine was noted to be acutely elevated to 2.78 mg/dL prior to starting treatment with brentuximab vedotin, a CD30 antibody-drug conjugate. CT scan of the abdomen also revealed bilateral enlargement of the kidneys (from 11 to 14 cm) not associated with hydronephrosis compared to a CT scan two months ago (Figure 1). The patient received his dose of brentuximab despite these findings and was referred to the Nephrology department several days later for progressive renal insufficiency.

When seen by the Nephrology department, he was reporting a several-day history of fevers, nausea, and vomiting. He denied NSAID use, gross hematuria, dysuria, or decreased urinary frequency. Physical exam was remarkable only for dry mucous membranes with stable vital signs. Labs were notable for creatinine of 3.42 mg/dL, BUN of 32 mg/dL, and white blood cell count of 10,900 with normal eosinophils. Urate, lactate dehydrogenase, and phosphorus were all normal at 4.3 mg/dL, 195 U/L, and 3.8 mg/dL, respectively. Urinalysis revealed <1 eosinophil, 4+ WBC, and 30+ protein. Urine : protein creatinine ratio was 0.76. The patient was admitted to the hospital for intravenous fluids and for kidney biopsy. Carnivora was again discontinued during this hospitalization.

**Figure 2:**
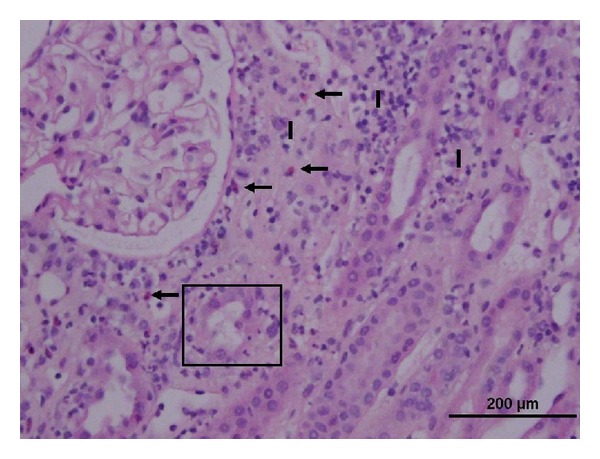
Photomicrograph showing active interstitial nephritis with acute tubulitis and mixed interstitial inflammation including numerous eosinophils. I: interstitial inflammation, arrows: eosinophils, and box: active tubulitis (H&E 30x magnification).

**Figure 3:**
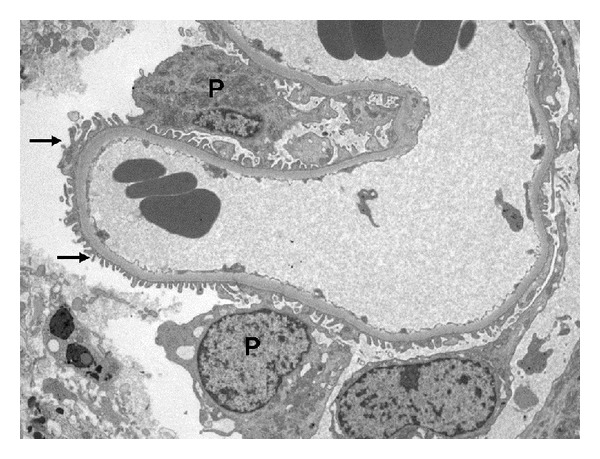
Electron microscopy showing lack of podocyte effacement or glomerular basement membrane changes. Arrows: intact foot processes, P: podocytes (original mag. = 3500x).

His kidney biopsy revealed acute tubulointerstitial nephritis with no evidence of glomerular injury or fibrosis. Light microscopy revealed diffuse interstitial inflammation with edema, which was predominantly lymphocytic with occasional plasma cells, polymorphic leukocytes, and frequent prominent clusters of eosinophils. Moderately extensive cortical tubular necrosis and injury with some dilated tubules were noted (Figure 2). Immunofluorescence was 1+ to 3+ positive for C3 in the arterioles and mesangium, tubular basement membranes, and Bowman's capsule. Immunofluorescence was negative for IgA, IgG, IgM, kappa, lambda, C1q, albumin, and fibrin. Electron microscopy showed the glomerular basement membranes were intact. He was treated with intravenous methylprednisolone 250 mg for 3 days, followed by a slow steroid taper. Creatinine improved to 2.99 mg/dL at discharge and continued to decline as an outpatient. The patient has not resumed Carnivora and creatinine has since remained stable at ~1.5 mg/dL (Figure 3).

## 3. Discussion

This case represents a previously unreported association of AIN in a patient using Carnivora extract. When considering the differential in this case, some considerations included uric acid nephropathy from tumor lysis syndrome, renal vein thrombosis, and obstruction from an enlarged lymph node or tumor. These diagnoses were effectively excluded through CT scan of the abdomen and phosphorus, urate, potassium, and calcium levels. Lymphomatous renal infiltration, seen in one-third of lymphoma patients on autopsy, was also a strong possibility due to bilateral enlargement on CT scan. However, few of these patients develop renal failure with only 12 reported cases in the literature [6, 7]. The absence of atypical lymphoid cells and prominent eosinophils on his renal biopsy argues against this process. Finally, ampicillin-sulbactam, promethazine, metoclopramide, and baclofen in the previous month are possible culprits; however they did not fit the typical temporal relationship as well as Carnivora and were given in limited doses. The reintroduction of Carnivora at a higher dose also makes it the more likely triggering factor. Finally, the discontinuation of Carnivora, along with initiation of steroids, did lead to improvement of his renal function making this the more likely cause. Penicillins such as ampicillin-sulbactam have been associated with AIN; however, there have been no reports of AIN with promethazine, metoclopramide, and baclofen.

The mechanism of AIN is not completely understood. One proposed mechanism is that absorption of various plasma proteins and molecules by tubular cells causes secretion of chemotactic and inflammatory mediators in the interstitium. Nuclear factor-kappaB (NF-kappaB) is a protein complex that regulates DNA transcription and upregulates inflammatory mediators and is overexpressed in the kidneys of proteinuric animals [8–14]. Increased trafficking of protein has been seen to upregulate RANTES production which is a chemoattractant molecule stimulated by NF-kappaB [15]. The inhibition of NF-kappaB has been shown to reduce cortical tubulointerstitial injury in rat models [16]. Carnivora is marketed as an immune suppressant primarily due to a compound plumbagin in the product that inhibits factor-kappaB (NF-kappaB) in lymphocytes [17]. Based on these findings, Carnivora would presumably decrease the incidence of interstitial nephritis. However, Carnivora is also largely composed of a variety of amino acids which when absorbed by the tubular cells can upregulate transcription of NF-kappaB and RANTES and stimulate an inflammatory reaction [18, 19]. Therefore, the components of Carnivora can both suppress and incite inflammation within the renal interstitium. This is similar to another case report of creatine, a high amino acid supplement, as the cause of interstitial nephritis in one patient; however this correlation is currently under debate [20].

Therefore, this case illustrates the potential for acute interstitial nephritis in a patient taking Carnivora and how early discontinuation of this medication can improve kidney recovery. The mechanism by which acute interstitial nephritis develops may similarly occur for other high protein supplements.
